# The etiology of resilience to disadvantage

**DOI:** 10.1002/jcv2.12033

**Published:** 2021-09-24

**Authors:** Alexandra Y. Vazquez, Elizabeth A. Shewark, D. Angus Clark, Kelly L. Klump, Luke W. Hyde, S. Alexandra Burt

**Affiliations:** ^1^ Department of Psychology Michigan State University East Lansing Michigan USA; ^2^ Department of Psychiatry University of Michigan Ann Arbor Michigan USA

**Keywords:** adversity, resilience, twins

## Abstract

**Background:**

Although early life exposure to chronic disadvantage is associated with deleterious outcomes, 40%–60% of exposed youth continue to thrive. To date, little is known about the etiology of these resilient outcomes.

**Methods:**

The current study examined child twin families living in disadvantaged contexts (*N* = 417 pairs) to elucidate the etiology of resilience. We evaluated maternal reports of the Child Behavior Checklist to examine three domains of resilience and general resilience.

**Results:**

Genetic, shared, and nonshared environmental influences significantly contributed to social resilience (22%, 61%, 17%, respectively) and psychiatric resilience (40%, 28%, 32%, respectively), but academic resilience was influenced only by genetic and nonshared environmental influences (65% and 35%, respectively). These three domains loaded significantly onto a latent resilience factor, with factor loadings ranging from 0.60 to 0.34. A common pathway model revealed that the variance common to all three forms of resilience was predominantly explained by genetic and non‐shared environmental influences (50% and 35%, respectively).

**Conclusions:**

These results support recent conceptualizations of resilience as a multifaceted construct influenced by both genetic and environmental influences, only some of which overlap across the various domains of resilience.

**Key points**

- Historically, research on the origins of resilient outcomes has focused on environmental influences. However, recent studies have begun to examine biological and genetic influences as well.
- While extant studies demonstrate that domains of resilience are differentially heritable, their imprecise measurement of resilience does not adequately discriminate between resilience and vulnerability. What's more, these studies are limited by their examination of only one or two domains of resilience.
- The current study was the first to examine the etiology of academic, social, psychiatric, and overall resilience in a high‐risk sample.
- Findings highlight the necessity of considering *both* biological and environmental contributions to domain‐specific and general resilience.

## INTRODUCTION

Disadvantage refers to a spectrum of circumstances emanating from systemic and societal inequities, and includes experiences such as low socio‐economic status (SES), limited access to resources (e.g., grocery stores), and/or exposure to community violence (e.g., Wodtke et al., 2011). Disadvantage has been consistently associated with poor health (Alvarado, 2016), poor academic performance (Duncan & Murnane, 2011; Wodtke et al., 2011), and behavioral problems (Campbell et al., 2000; Winslow & Shaw, 2007), outcomes that can themselves perpetuate systemic inequity across generations (Duncan & Murnane, 2011). Despite these robust associations, however, it is equally true that not all youth residing in disadvantaged contexts evidence maladaptive outcomes. Indeed, prior work has indicated that roughly 40%–60% of exposed youth evidence resilient outcomes (Luthar, 2006; Masten, 2001; Vanderbilt‐Adriance & Shaw, 2008), or successful adaption and competent functioning in the face of adversity (Luthar et al., 2000; Masten, 2001; Rutter, 2006). What accounts for these adaptive outcomes in the face of adversity? Although research has historically focused on environmental predictors of resilience, recent scientific advances have enabled resilience researchers to begin to illuminate the role of biological and genetic influences. The current study aimed to augment recent literature by simultaneously examining both the environmental and genetic contributions to variability in resilience to disadvantage.

Recent conceptualizations of resilience highlight its dynamism (Luthar et al., 2015; Masten et al., 2021) and posit that it can be understood as both a process and a multifaceted outcome (Miller‐Graff, 2020). Key domains of this multifaceted outcome include both psychiatric resilience and the presence of specific competencies, most notably, academic resilience and social resilience. Interestingly, some youth show resilience across multiple domains (Masten & Curtis, 2000), while others demonstrate only domain specific resilience (Luthar et al., 2000). It is therefore necessary to both separately assess these specific domains and examine the extent to which general resilience develops.

Researchers have historically examined resilience via the ecological transactional model (Cicchetti & Lynch, 1993; Felner & DeVries, 2013), which emphasizes the importance of culture, community, family, and ontogenetic development in our understanding of the development of resilience. The model posits that risk (e.g., neglect) and protective (e.g., parental warmth) factors across levels of ecology influence one another. More recently, the biopsychosocial framework has expanded on the ecological transactional model (Feder et al., 2019), highlighting interactions between genes and the environment and incorporating a consideration of epigenetics, neural circuity, individual factors (e.g., self‐efficacy), and interventions (e.g., youth developmental programs). Critically, although there has been a notable recent focus on the neurobiology of resilience (Cathomas et al., 2019; Murrough & Russo, 2019; Osório et al., 2017), behavioral genetic research on the etiology of resilience has remained scarce.

### How behavioral genetics might inform our understanding of resilience

Behavioral genetics research can uniquely augment extant literature by jointly examining genetic and environmental influences on resilience. Behavioral genetics methods leverage the genetic similarity between individuals (e.g., twins and adoptees) to parse the variance of a construct into additive genetics (A; i.e., the effect of genes summed over loci), shared environmental (C; i.e., the environment shared by twins growing up in the same family; e.g., parenting style), and nonshared environmental (E; i.e., the environment not shared by twins raised in the same family; e.g., peer relationships) influences.

Despite its promise, there are few twin studies on the etiology of resilient outcomes (Amstadter et al., 2014; Boardman et al., 2008; Kim‐Cohen et al., 2004). Kim‐Cohen et al. (2004), for example, examined 1116 5‐year‐old twin pairs oversampled for socio‐economic disadvantage and found that psychiatric “resilience” (specifically, an absence of antisocial behavior) was largely heritable, whereas cognitive “resilience” was influenced by genetic and environmental factors. Amstadter et al. (2014) and Boardman et al. (2008) similalry found evidence of genetic and nonshared environmental influences on psychiatric “resilience” in adults.

Although these studies represent an important first step in the study of psychiatric resilience, they suffer from a few key limitations. First, they have restricted their outcomes to only one or two domains of resilience, with no consideration of social resilience or general resilience. Second, none of these studies focused on those families experiencing substantial adversity. Instead, “resilience” was operationalized as any deviation (either positive or negative) from the linear prediction of a given outcome by adversity (e.g., SES). As a consequence, and as noted by Kim‐Cohen et al., their operationalization of resilience necessarily included vulnerability in the absence of adversity as well as resilience in the presence of adversity. This is an important point, as the etiology of vulnerability and resilience seem apriori likely to differ, which accordingly may cloud etiologic estimates of resilience in particular. Similarly, the residualized approach is less than ideal in that error in the measurement of an outcome could also be considered “resilience” in this approach (Newsome & Sullivan, 2014).

The current study sought to address these limitations, examining a unique dataset of twins exposed to at least two of three sources of disadvantage (i.e., family poverty, neighborhood poverty, and community violence). We examined the etiology of resilience to disadvantage within domains (i.e., academic, social, and psychiatric) and general resilience across domains (modeling a latent construct of resilience).

## METHODS

### Participants

The Twin Study of Behavioral and Emotional Development in Children is a part of the Michigan State University Twin Registry (Burt & Klump, 2013, 2019; Klump & Burt, 2006) and includes a population‐based (*N* = 528 families) and an “at‐risk” sample (*N* = 502 families). Families were recruited from across Lower Michigan between 2008 and 2015 directly from birth records or from the Michigan Twins Project registry. Families were mailed anonymous recruitment mailings in conjunction with the Michigan Department of Health and Human Services. Recruitment for the “at‐risk” sample was identical, except that it was restricted to those families residing in neighborhoods where at least 10.5% of households were below the 2008 poverty line, the mean level of poverty in Michigan at the time the study began (for detailed information see Burt & Klump, 2019). To be eligible for participation in the TBED‐C, neither twin could have a cognitive or physical condition that would preclude completion of the assessment (as assessed via parental screening; e.g., a significant developmental delay). Children provided informed assent, and parents provided informed consent for themselves and their children.

Participants were considered to be experiencing substantial “disadvantage” and therefore included in the current study if they met criteria for two of three indicators: family poverty, neighborhood poverty, and exposure to community violence (as used in Burt et al., 2018). Family poverty was measured using maternal reports of total family income; those families with a combined income of $55K or less (below the living wage in the state at that time of assessment) met criteria for this indicator. Mean family income in these data was $30,000–$35,000 (vs. $72,027 in the population‐based sample). Neighborhood poverty was assessed using Census data; participants residing in Census tracts where 20% or more of households were below the 2008 poverty line met criteria for this indicator. Mean neighborhood poverty level in these data was 27%, compared to 11.4% in the population‐based sample. Finally, exposure to community violence was assessed via maternal reports on the indirect violence scale of the KID‐SAVE (i.e., “witnessing less severe interpersonal violence or hearing about violent events”; Flowers et al., 2000). Participants who endorsed 30% or more of the items on this scale met criteria for this indicator (15% in the current sample and 7% in the population‐based sample).

Of note, less than half of participants who met criteria for the family poverty indicator (45.8%) also met criteria for the neighborhood poverty indicator. Similarly, family poverty and neighborhood poverty were respectively correlated −0.15 (*p* < .05) and 0.18 (*p* < .05) with community violence, indicating that our three indicators of disadvantage function as at least partially independent experiences of disadvantage. In total, 417 twin pairs (monozygotics [MZs]: 156 and dizygotics [DZs]: 261) were experiencing at least two of the three forms of disadvantage and were therefore included in analyses. Due to small amounts of missing data for the outcomes assessed, the analytic sample sizes range from 151 to 155 MZ pairs and 254 to 260 DZ pairs.

All twins ranged in age from 6 to 11 years old (Mean = 7.91, SD = 1.47) at the time of their participation. Participants primarily identified as White (68.5%), 19.9% identified as Black, 1.9% identified as Native American, and 9.7% identified as mixed race or a race/ethnicity predominant in less than 1% of the sample (i.e., Asian, Latinx, or Pacific Islander).

### Measures

Twin zygosity was determined via physical similarity questionnaires (which have demonstrated over 95% accuracy) administered to the twins' primary caregiver (Peeters et al., 1998). Using this scale, 156 pairs were identified as monozygotic (MZ; 85 male–male and 71 female–female), and 261 were identified as DZ (90 male–male, 82 female–female, and 89 male–female).

#### Resilience

The Achenbach Child Behavior Checklist (CBCL; Achenbach & Rescorla, 2001) is one of the most commonly used instruments for assessing academic and social competence, as well as internalizing and externalizing problems prior to adulthood (Nakamura et al., 2009). Mothers rated the extent to which a series of statements described each twin's behavior during the past 6 months; most responses were made on a 3‐point scale ranging from 0 (never) to 2 (often/mostly true).

The School Competency subscale of the CBCL (*α* = .58) served as our measure of academic resilience. This scale assesses school performance across subject domains, special education services received, repeated classes, and academic or other school related problems (e.g., Does your child receive special education or remedial services?). The Social Competency subscale of the CBCL (*α* = .51) served as our measure of social resilience. This scale assesses the child's involvement in organizations, number of friends, contact with friends, behavior with others, and behavior alone (e.g., About how many times a week does your child do things with any friends outside of regular school hours?). Of note, low internal consistency is often seen in multidimensional scales (Schmitt, 1996), which may explain the lower values reported for competency subscales in the current sample. For both domains, higher scores are indicative of better functioning or “more” resilience.

To measure psychiatric resilience (*α* = .77), we examined the eight psychopathology subscales from the CBCL: Anxious/Depressed (e.g., Fears certain animals, situations, or places, other than school), Withdrawn/Depressed (e.g., There is very little he/she enjoys), Somatic Complaints (e.g., Constipated, does not move bowels), Social Problems (e.g., Complains of loneliness), Thought Problems (e.g., Hears sounds or voices that are not there), Attention Problems (e.g., Cannot concentrate, cannot pay attention for long), Rule‐Breaking (e.g., Breaks rules at home, school, or elsewhere), and Aggressive Behavior (e.g., Destroys things belonging to his/her family or others). We first recoded each scale as a dichotomous variable that indicated whether the child was at or above (coded 0) the borderline clinically significant range for that subscale (Achenbach & Rescorla, 2001) or was in the normative range (i.e., below the borderline cut‐point for that subscale; coded 1). The eight dichotomous variables were then summed to serve as our psychiatric resilience indicator, ranging from 0 to 8 (where 8 indicates a lack of psychopathology on any scale). Because psychiatric resilience was not normally distributed, we log‐transformed this variable for analyses.

While sex and age differences have been found in constructs related to the domains of resilience examined (e.g., social competence and psychopathology; Ford, 1982; Hofstra & Verhulst, 2000; Solomon & Herman, 2009), exploring these differences is outside of the scope of the current study and thus we regressed both out of all variables. We then created standardized residuals for use in the formal model fitting analyses.

### Statistical analyses

Prior to fitting models, we examined descriptive statistics as well as twin intraclass correlations for each domain of resilience. Our statistical analyses involved two parts. First, we estimated separate univariate ACE models for social, academic, and psychiatric resilience (Prescott, 2004). The univariate ACE model decomposes the variance of a given phenotype into additive genetic (A), shared (C), and non‐shared environmental (E) variance components. Second, the common pathway model was estimated to examine the ACE contributions to an overall latent resilience factor. In this model, social, academic, and psychiatric resilience were specified as indicators of a common resilience factor that captured the covariation across all three indicators. Both the common resilience factor and the indicator specific residual variances were then decomposed into additive genetic, shared, and non‐shared environment components. Note that all measurement error is necessarily contained within the variable‐specific E estimate, since unsystematic error cannot covary across phenotypes. An independent pathway model, in which there is no phenotypic common factor, was estimated for comparison.

Models were evaluated on their absolute fit indices (CFI (≥0.90), RMSEA, (≤0.07), SRMR (≤0.07), *X* ^2^ (*p* > .05)), and the common and independent pathway models were also evaluated on their comparative fit indices (SBIC, AIC, BIC; lower values indicate better fit). Models were esimated in Mplus version 8.3 (Muthen & Muthen, 1998–2017) using full information maximum likelihood estimation (Lang & Little, 2018), although twin pairs with missing data on all variables were excluded by Mplus (Ns for the final models are presented in Table 2). Confidence intervals were derived using nonparametric percentile bootstrapping (with 10,000 draws), which provides reliable confidence intervals for assessing parameter estimate precision under a variety of complex data conditions without concerns for violating the typical assumptions of structural equation models (Falk, 2018).

## RESULTS

Levels of social and academic resilience were typically moderate, while the rate of psychiatric resilience was high (see Table 1). There were significant, moderate phenotypic correlations between social and academic resilience (*r* = .18, *p* < .05), social and psychiatric resilience (*r* = .20, *p* < .05), and academic and psychiatric resilience (*r* = .30, *p* < .05). We also computed twin intraclass correlations (see Table 1). The correlations between MZ twins were significantly higher than those for DZ twins for all three domains of resilience. Such findings pointed to probable additive genetic influences. The MZ correlation was not double that of the DZ correlation for either social or psychiatric resilience, however, pointing to probable shared environmental influences on those phenotypes as well.

**TABLE 1:** Descriptive statistics

Construct	Monozygotic twins (MZ)							Dizygotic twins (DZ)						
	*N*	Mean	SD	Scale range	Min	Max	Intraclass correlation	*N*	Mean	SD	Scale range	Min	Max	Intraclass correlation
Social resilience	154	7.27	2.40	0–15	1.5	12.50	.84	257	6.89	2.35	0–15	0	13.50	.73
Academic resilience	151	4.71	1.17	0–6	0	6	.70	254	4.70	1.06	0–6	0	6	.21
Psychiatric resilience	155	7.35	1.28	0–8	1	8	.62	260	7.09	1.59	0–8	1	8	.50

*Note*: For each resilience domain, higher scores are indicative of better functioning or “more” resilience. All MZ intraclass correlations are significantly different from the DZ intraclass correlations for the corresponding measure.

Abbreviations: DZ, Dizygotic; max, maximum; min, minimum; MZ, monozygotic; *N*, number of twin pairs; SD, standard deviation.

### Biometric model‐fitting results

**FIGURE 1:**
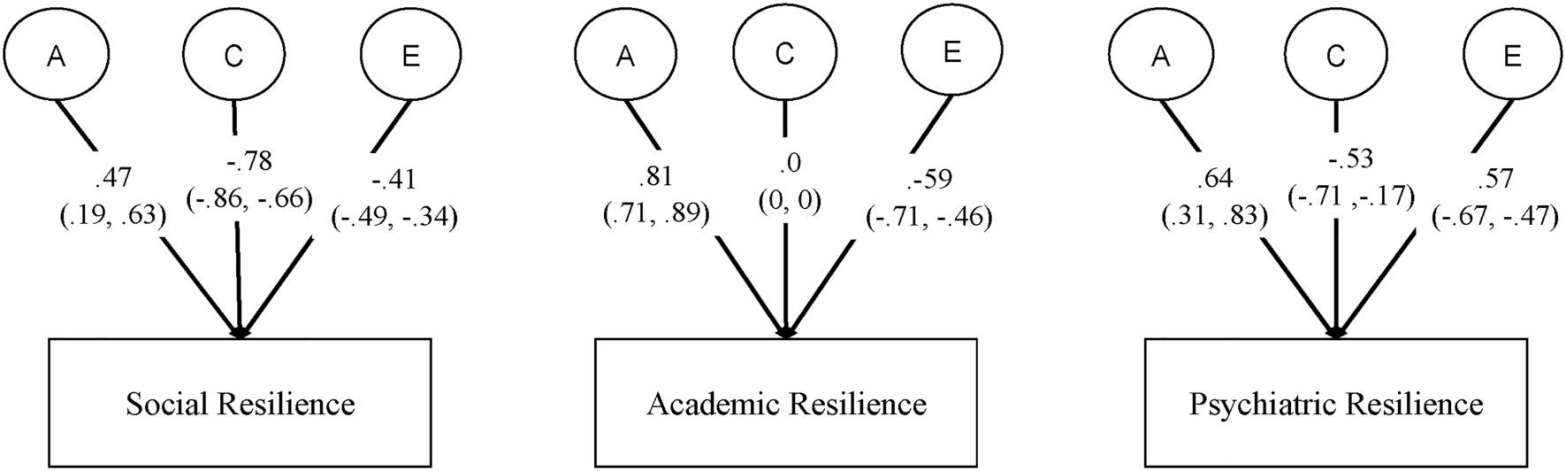
Univariate results for social, academic, and psychiatric resilience. Standardized path coefficients are reported. Path coefficients can be used to calculate variance (e.g., −0.78^2^ = 61%). 95% Confidence intervals are presented in parentheses

Univariate model results are presented in Table 2; standardized path coefficients are depicted in Figure 1. As seen there, variability in social resilience appeared to be a function of genetic, shared, and non‐shared environmental variance (i.e., A = 22%, C = 61%, E = 17%), whereas variability in academic resilience was influenced only by additive genetic and non‐shared environment variance components (i.e., A = 65%, E = 35%). Variability in psychiatric resilience was similarly influenced by all three variance components (i.e., A = 40%, C = 28%, E = 32%).

**TABLE 2:** Model fit indices

Fit statistics	Resilience domain univariate ACE models			IPMs	Latent resilience factor CPMs			
	Academic resilience	Psychiatric resilience	Social resilience	ACE	ACE	AE	CE	E
Log likelihood (H0/H1)	−1095.97/−1092.76	−1102.42/−1097.68	−982.86/−972.37	−3113.57/−3085.92	−3115.57/−3085.92	−3115.77/−3085.92	−3117.45/−3085.92	−3124.80/−3085.92
AIC	2199.94	2212.84	1973.71	6269.15	6265.13	6263.53	6266.89	6285.61
BIC	2216.05	2228.98	1989.82	6353.84	6333.70	6328.06	6331.42	6358.20
Adjusted BIC	2203.36	2216.28	1977.13	6287.21	6279.75	6277.29	6280.65	6301.08
*X* ^2^(DF)	6.42(6)	9.48(6)	20.98(6)	55.30(33)	59.29(37)	59.69(38)	63.05(38)	77.76(36)
RMSEA	0.02	0.05	0.11	.06	.05	.05	.06	.08
CFI	1.00	0.98	0.96	.97	.97	.97	.97	.95
SRMR	0.07	0.11	0.09	.08	.08	.08	.08	.10
A	.65 (.50, .79)	.40 (.10, .70)	.22 (.04, .40)	‐	.50 (0, .86)	.65 (.32, .88)	‐	‐
C	0 (0, 0)	.28 (.03, .51)	.61 (.46, .75)	‐	.15 (0, .59)	‐	.52 (.25, .72)	‐
E	.35 (.21, .50)	.32 (.22, .45)	.17 (.11, .24)	‐	.35 (.12, .67)	.35 (.12, .68)	.48 (.28, .75)	1.00 (1, 1)

*Note*: A, C, and E are squared parameter estimates. See Supplemental Figure S1 for the IPM results.

Abbreviations: AIC, Akaike's information criterion; BIC, Bayesian information criterion; CFI, Comparative Fit Index; CPMs, Common Pathway Models; H0/H1, Null hypothesis/alternative hypothesis; IPM, Independent Pathway Model; RMSEA, Root Mean Square Error of Approximation; SRMR, Standardized Root Mean Square Residual; *X* ^2^(DF), Chi‐square (Degrees of Freedom).

**FIGURE 2:**
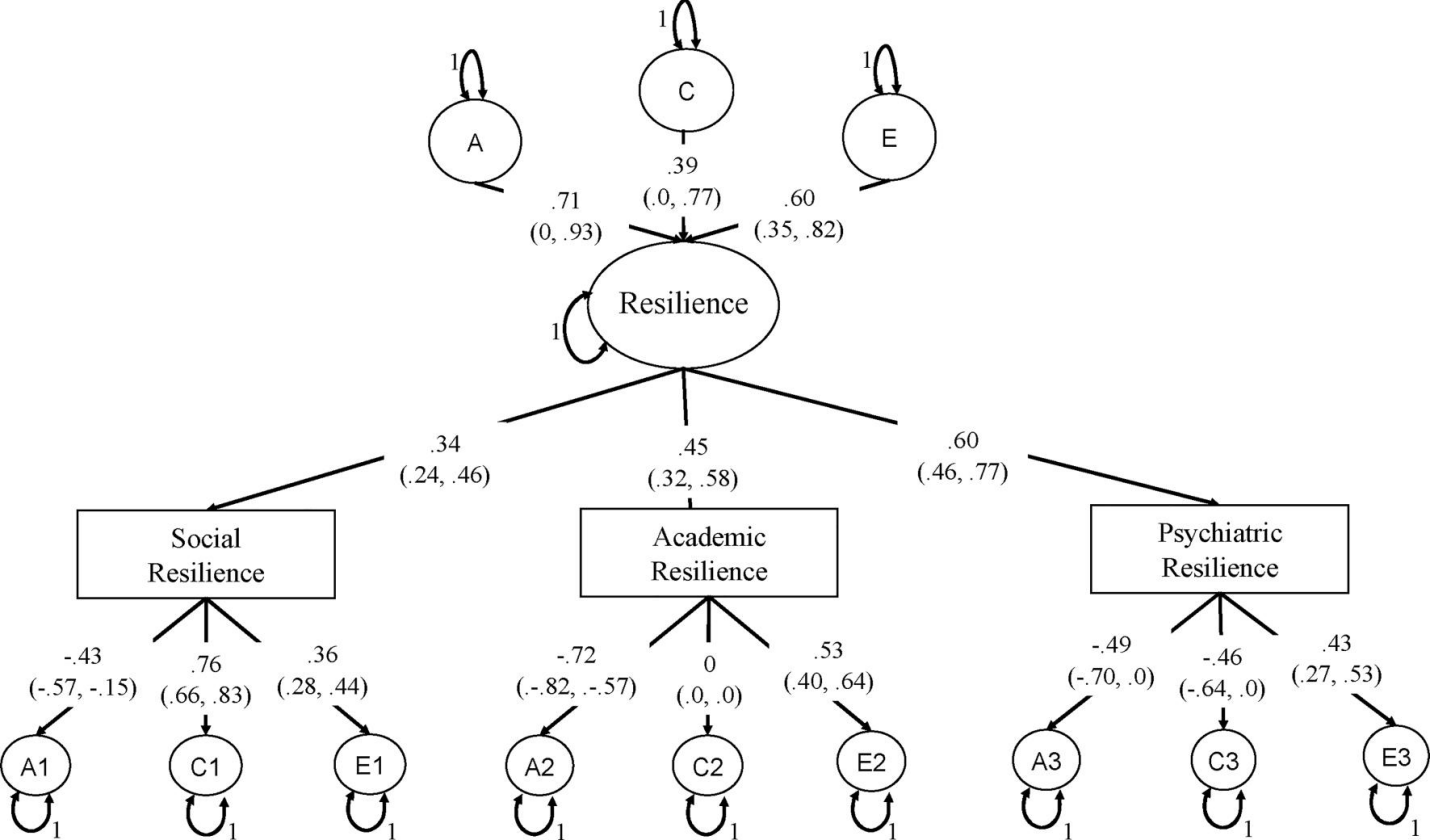
Resilience common factor model results. Standardized path coefficients are reported. Confidence intervals are presented in parentheses

The common pathway model evidenced better fit than the independent pathway model (Table 2); parameter estimates for the common and independent pathway models are presented in Figures 2 and S1 (see Supporting Information), respectively. Regarding the common pathway model, the E indicator‐specific residual variances were uniformly larger than zero, consistent with the fact that this component of variance contains all unsystematic measurement error for that phenotype. However, we also observed unique A and C influences specific to social resilience, and unique A influences on academic resilience.

The three domains of resilience also loaded significantly onto our latent resilience factor, with factor loadings ranging from 0.60 for psychiatric resilience to 0.34 for social resilience. Decomposition of the latent factor into its genetic and environmental components further revealed that 50% of common variance was accounted for by genetic influences, 15% by the shared environment, and 35% by the non‐shared environment, although only the latter was statistically significant. We thus compared the full ACE common pathway model to reduced models (models with either AC, AE, CE, or E on the latent factor were all considered; see Table 2) in a series of post‐hoc analyses. Results indicated that the best‐fitting model was the AE model as evidenced by marginally lower log likelihood, AIC, BIC, and adjusted BIC values, although the A estimate remained nonsignificant (A = 65%, E = 35%).

## DISCUSSION

The goal of this study was to elucidate the etiology of resilience to disadvantage across multiple domains using a sample of 417 twin pairs exposed to disadvantage. Univariate results revealed that social resilience was predominantly explained by shared environmental influences, academic resilience was predominantly explained by genetic influences, and psychiatric resilience was relatively equally explained by genetic and environmental influences. A common pathway model further revealed that a portion of these genetic and environmental influences were common across all three domains of resilience, while some were domain‐specific.

Such findings are consistent with early conceptual models in showing that, although they are separable, the various domains of resilience are also related to one another (Kaplan, 2013; Masten & Curtis, 2000). The common pathway model indicated that social, academic, and psychiatric resilience all cohere to form a latent factor of resilience, illustrating the importance of assessing both domain specific and global indicators of resilience. Moreover, the variance of this latent resilience factor was explained by both genetic and non‐shared environmental factors, supporting the biopsychosocial model of resilience (Feder et al., 2019). While these findings support more recent conceptualizations of resilience as a multifaceted construct, they also suggest that there are shared etiologic processes underlying all domains of resilience.

That said, our results also suggest that there are domain‐specific processes as well. Shared environmental influences appear to play a key role in enabling social resilience and to a lesser extent, psychiatric resilience (Burt, 2009). Such findings are consistent with past research pointing to the importance of family (e.g., parental warmth) and community‐level factors (e.g., social cohesion) (Roisman & Fraley, 2012). In comparison, genetic factors were the largest influence for academic resilience (Edelbrock et al., 1995; Hudziak et al., 2003) though they were also important in social resilience (Edelbrock et al., 1995).

In sum, our results advance prior work in two ways. First, while our results are consistent with prior theory and empirical data pointing to the presence of domain‐specific resilience (e.g., Luthar et al., 2000), our results also highlight the presence of etiologic processes, both genetic and environmental, that influence resilience more generally. Second, our findings provide support for the biopsychosocial model's contention that both biology and environmental influences play a key role in the development of resilience. Future studies should seek to identify biological processes and environmental experiences that contribute to general versus domain‐specific resilience.

Of note, our findings were only partially consistent with prior twin studies of “resilience.” Kim‐Cohen et al., for example, found that psychiatric “resilience” (specifically, an absence of antisocial behavior) was largely heritable, whereas cognitive “resilience” was influenced by genetic and environmental factors. As noted, however, all prior twin studies operationalized resilience on relative terms (difference scores of predicted vs. actual functioning), such that “resilient youth” were simply those who exhibited better functioning than predicted based on SES deprivation and/or stressful life events but not necessarily *adaptive* levels of functioning (Amstadter et al., 2014; Boardman et al., 2008; Kim‐Cohen et al., 2004). What's more, individuals who demonstrated vulnerability in the absence of risk were included in their operationalization of “resilience” (Amstadter et al., 2014; Boardman et al., 2008; Kim‐Cohen et al., 2004). By contrast, our sample was directly assessed for disadvantage and families were only included in these analyses if they had experienced at least two indicators of chronic disadvantage (i.e., family poverty, neighborhood poverty, and exposure to community violence). The current study is thus the first to evaluate resilience in an exclusively high‐risk twin sample. As such, we would argue that our approach better captured the multifaceted concept of adaptive competency in the face of adversity that is core to modern‐day conceptualizations of resilience.

### Limitations

There were several limitations to note. First, this study was restricted to maternal reports on a single measure. This is an important limitation, as the examination of multiple informants or measures would likely capture a more complete view of children's functioning. Future studies should aim to measure multiple indicators of functioning within each domain of resilience. Second, while the “at‐risk” sample used in this study is more racially diverse than our population‐based sample, it remains predominantly White, limiting the generalizability of our findings to other racial and ethnic populations. Future studies should make explicit efforts to collect more racially and ethnically diverse samples. Third, while this study focused specifically on resilience to environmental disadvantage, there are other forms of resilience (e.g., resilience to abuse) that can and should be examined, as it is possible that the etiology of other forms of resilience could vary from that reported here.

Finally, although the A and C parameters were not statistically significant in the common pathway models, each accounted for a sizable amount of variance. To better understand these results, we conducted a brief Monte‐Carlo power analysis. Across 1000 replications, we were only able to detect 50% of the additive genetic variance. In addition, our ability to detect the shared environment variance components was low (9%), which is consistent with past work indicating that studies are often underpowered to detect C (Burt, 2009). Such results imply that the current sample size may be inadequate to reliably detect additive genetic and shared environmental effects of this magnitude on a latent factor like that examined here. However, these results, together with the magnitude of the A path estimate (0.71) and the model comparison results (which tend to have more power due to fewer parameters being estimated; Kline, 2011), suggest that the genetic signal on the latent factor is unlikely to be statistical noise, though it will need to be confirmed in larger samples.

## CONCLUSION

This study is the first to examine the etiology of resilience both within and across domains in a high‐risk sample. In doing so, we were able to examine the underlying etiology of general resilience while also exploring etiological differences across academic, social, and psychiatric domains of resilience. Our results revealed differential etiologies across domains, thereby supporting the conceptualization of resilience as a multifaceted construct and the need to examine domains separately. However, we also found that academic, social, and psychiatric resilience each contribute to a latent factor of resilience which is explained by both genetic and environmental influence. Such findings suggest that, while partially separable phenotypically and etiologically, the various domains of resilience are influenced by core etiologic processes common to all domains. Collectively, these findings strongly suggest that in order to fully understand the processes that support resilience, we must simultaneously consider environmental and biological influences, and we must do so at both domain‐specific and overall levels.

## CONFLICT OF INTEREST

The authors have declared that they have no competing or potential conflicts of interest.

## ETHICS STATEMENT

The institutional review board at Michigan State University approved all procedures in this study in accordance with ethical standards.

## AUTHOR CONTRIBUTIONS

Alexandra Y. Vazquez: Conceptualization; Formal analysis; Methodology; Writing‐original draft; Writing‐review & editing. Elizabeth A. Shewark: Conceptualization; Formal analysis; Methodology; Writing‐original draft; Writing‐review & editing. D. Angus Clark: Formal analysis; Methodology; Writing‐review & editing. Kelly L. Klump: Data curation; Funding acquisition; Investigation; Methodology; Project administration; Writing‐review & editing. Luke W. Hyde: Conceptualization; Writing‐review & editing. S. Alexandra Burt: Conceptualization; Funding acquisition; Investigation; Methodology; Project administration; Supervision; Writing‐review & editing.

## Supporting information

Figure S1

## Data Availability

The data that support the findings of this study are available on request from the corresponding author. The data are not publicly available due to privacy or ethical restrictions.
