# Supplementary material for: The etiology of resilience to disadvantage
Source: JCPP Adv. 2021 Sep 24;1(3):e12033. doi: 10.1002/jcv2.12033 (PMC8890479; doi:10.1002/jcv2.12033)
Supplement: Supplementary file 1 — Figure S1 [file JCV2-1-e12033-s001.docx]

**Supporting Information**

Figure S1.  Resilience independent pathway model results

*Note.* Standardized path coefficients are reported. Confidence intervals are presented in parentheses. Ac, Cc, and Ec refer to the common ACE factors, whereas A_1_ C_1_ E_1_ are specific to each domain.
